# Design of Readout Circuit with Quadrature Error and Auxiliary PLL for MEMS Vibratory Gyroscope

**DOI:** 10.3390/s20164564

**Published:** 2020-08-14

**Authors:** Hua Chen, Yanqing Zhong

**Affiliations:** Smart Sensing Center, Institute of Microelectronics, Chinese Academy of Sciences, Beijing 100029, China; zhongyanqing@ime.ac.cn

**Keywords:** coherent demodulation, quadrature error, phase alignment, phase-locked loop (PLL), MEMS gyro, vibratory gyroscopes, MEMS sensors

## Abstract

Traditional MEMS gyroscope readout eliminates quadrature error and relies on the phase relationship between the drive displacement and the Coriolis position to accomplish a coherent demodulation. This scheme shows some risk, especially for a mode-matching gyro. If only a slight resonant frequency deviation between the drive and sense mode occurs, a dramatic change in the phase relationship follows, which leads to a wrong demodulation. To solve this, this paper proposes a new readout based on the quadrature error and an auxiliary phase-locked loop (PLL). By tuning the phase shifter in the sense-mode circuit, letting the quadrature error and the carrier of the mixer be in 90° phase alignment, the Coriolis was simultaneously in phase with the carrier. Hence, the demodulation was accomplished. The carrier comes from the PLL output of the drive-mode circuit due to its low jitter and independence of the work mode of the gyro. Moreover, an auxiliary PLL is used to filter the quadrature error to enhance the phase alignment accuracy. Through an elaborate design, a printed circuit board was used to verify the proposed idea. The experimental results show the readout circuit functioned well. The scale factor of the gyro was 6.8 mV/°/s, and the bias instability was 204°/h.

## 1. Introduction

Gyroscopes are important sensors in inertial navigation devices and global positioning systems. Traditional mechanical gyroscopes cannot meet the requirements of modern application systems due to their large size, heavy weight, high power, high price, low bandwidth and low shock resistance. In the later period, fiber optic gyroscopes and laser gyroscopes appeared, but the problems of high cost, large volume and high power were still not solved. These problems remained the bottleneck restricting their application and development. The capacitive silicon-based micromechanical gyroscope integrates the advantages of MEMS technology and integrated circuit technology. It has the advantages of low cost, small size, light weight and low power consumption (Low-CSWaP). It has been widely used in many fields such as the consumer, automotive, medical and industrial fields. With the continuous improvement of performance, it will excel in high-end applications such as navigation, defense, aviation and aerospace [1,2]. Recently, high performance gyros such as the frame type [3,4,5,6,7], the disc type [8,9,10,11,12], the butterfly [13,14,15,16,17,18,19], etc. have attracted a lot of attention from academia and industry. The vibratory MEMS gyro is based on the Coriolis effect to measure the input angular velocity [20]. However, due to the non-ideality of the micro-machine process, the direction of the drive displacement cannot be completely consistent with the drive axis, and a quadrature error is formed in the sense axis, which interferes with the Coriolis signal. Compared with the Coriolis signal, the amplitude of the quadrature error is very large.On the other hand, the gyro often works in the low-pass region to reduce sensitivity to environment fluctuations. Under this condition, the Coriolis signal is very weak. How to extract such a small signal from the huge interference has become an important research issue.

To address it, many studies eliminate the quadrature error and amplify the Coriolis signal. There are three methods: (1) use a special device structure or trimming process to prevent the drive vibration from deviating [21,22,23]; (2) set a quadrature error cancellation electrode at the sense axis and feed the quadrature error back to the electrode to offset the unwanted force [7,8,17,24,25,26]; (3) bring the quadrature error back to the input of the preamplifier and cancel the quadrature error in the electrical domain [27,28,29]. However, the first method increases the production time and cost, and cannot fix the quadrature error in real time considering long-term use and environmental change. The second method will not work for some special devices due to the difficulty of constructing the cancellation electrode. Even if it is performed successfully, additional noise is introduced on the electrode and pollutes the Coriolis force, which reduces the mechanical signal-to-noise ratio. The third method adds electrical noise directly to the input of the preamplifier, reducing the electrical signal-to-noise ratio. Besides, both of the latter increase the complexity of the readout circuit. 

As for preserving the quadrature error, Sharma et al. [30] utilized the quadrature error to build the mode-matching circuit and achieved a high performance readout. Norouzpour-shirazi et al. [31] took advantage of the large amplitude feature of the quadrature error, converted the amplitude modulation signal to a phase modulation signal, and used a digital demodulation to extract the angular rate information. Inspired by this, this paper proposes a novel readout based on the quadrature error and an auxiliary phase-locked loop (PLL). The quadrature error is used as an indicator while the PLL output of the drive mode is adopted as the carrier. By adjusting the phase shifter in the sense mode, the indicator is allowed to lag the carrier by 90°. Simultaneously, the Coriolis signal is in phase with the carrier, which leads to a correct coherent demodulation. The method does not depend on the relationship between the two resonant frequencies; that is, it is independent of the working mode of the gyro. To improve the phase alignment accuracy, the quadrature error is allowed to pass through an auxiliary PLL and the output is taken as the new indicator. This new readout is verified in a printed circuit board (PCB). The measurement results show the scale factor is 6.8 mV/°/s and the bias instability is 204°/h. Although the bias instability is two orders of magnitude larger than the latest research result [18], it is believed that with subsequent optimization, the performance will be greatly improved. The article is organized as follows. First, it briefly introduces the MEMS gyro device and then illustrates the new readout architecture in detail. Afterwards, the elaborated design of the drive/sense mode circuit is presented. Finally, it provides the experimental results and comes to a conclusion.

## 2. The MEMS Vibratory Gyro

**Figure 1:**
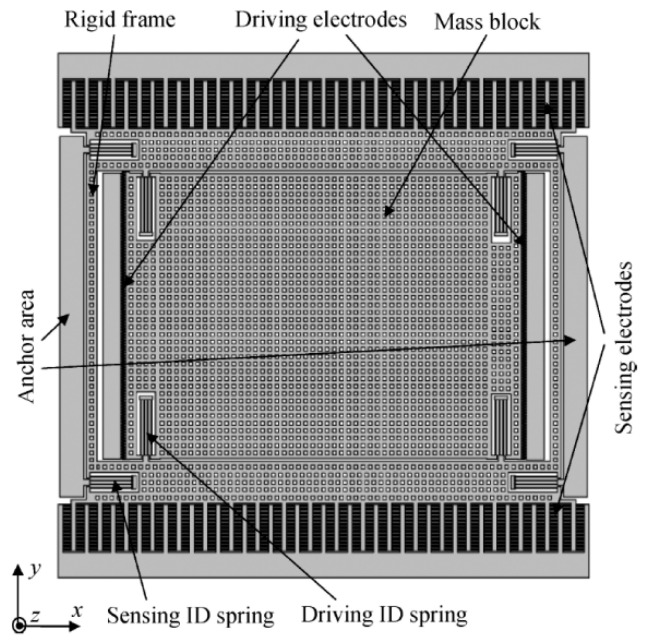
Diagram of the silicon on insulator (SOI) MEMS vibratory gyroscope [32].

The device is frame type, as shown in Figure 1, which is based on electrostatic excitation and capacitive detection transduction [32]. It is based on a silicon on insulator (SOI) process and fabricated by bulk micromachining technology. The thickness of the structure layer is as high as 100 μm, which produces a large elastic stiffness in the thickness direction and suppresses the out of plane vibration. With a large mass and large detection capacitance, the output current in the sense mode is relatively large, which is helpful for improving the mechanical sensitivity. The driving and detection electrodes of the drive mode adopt an interdigital comb structure, while the differential detection electrodes of the sense mode adopt the parallel plate style, which shows high sensitivity. The gyro was made in-house and wafer-level packaged with a 100 mTorr vacuum. The drive-mode resonant frequency was 5.817 KHz, and the sense-mode was 6.05 KHz. The drive-mode quality factor (Q) value was 35,000 while the sense-mode was 500. The Q value of the sense mode was low because of two points. (1) The anchor losses in the sense mode are much higher than those in the drive mode. (2) The electrode structure of the sense mode is a parallel plate instead of an interdigital comb; hence, the squeeze film damping is much larger than that in the drive mode. However, the low Qs value brings a benefit; that is, the amplitude and phase stability of the Coriolis displacement at the drive-mode resonant frequency are higher when working in the low-pass region. That is, the error introduced by frequency or damping changes is small. The parameters of the gyro are summarized in Table 1.

## 3. Proposed Readout Circuit Architecture

**Figure 2:**
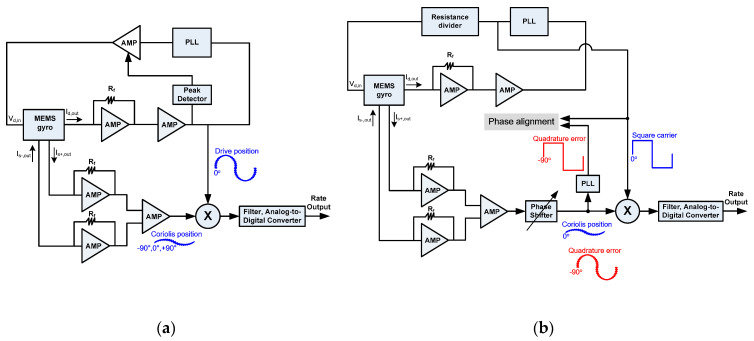
(**a**)Traditional readout circuit; (**b**) The proposed readout circuit based on quadrature error and auxiliary phase-locked loop (PLL).

The traditional readout circuit, as shown in Figure 2a, is based on the phase relationship between the drive displacement and the Coriolis [3,6,20]. In particular, in the mode matching condition, the drive displacement is anti-phase with the Coriolis and the readout circuit can perform well. However, when mode mismatch happens due to environmental disturbance, the Coriolis leads or lags the drive displacement by 90°, which leads to a wrong demodulation [20]. Besides, considering the different signal paths, a slight phase error between the drive displacement and the Coriolis exists. On the other hand, the two inputs of the mixer, which come directly from the output of the amplifier, contain some noise. Both of these two problems lower the demodulation performance.

This paper presents a new readout scheme, as shown in Figure 2b, which is based on the idea that by aligning the phase difference between the quadrature error and the carrier at 90°, the Coriolis is simultaneously in phase with the carrier, which leads to a correct demodulation. This method can demodulate the angular rate information correctly, regardless of the work mode of the gyro. The quadrature error is selected as the indicator due to its large amplitude and the feature of the constant 90° phase difference between the quadrature error and the Coriolis. The PLL output in the drive mode, instead of the drive displacement, is adopted as the carrier because of two points. (1) The jitter of the PLL output is low. (2) The PLL output can be viewed as just a carrier that does not stand for the drive displacement or velocity. This idea removes the limitation of the phase relationship between the drive displacement and the Coriolis. As depicted in Figure 2b, the phase shifter in the sense mode is a key block by which the 90° phase alignment can be achieved. The auxiliary PLL is another important module, with which the jitter of the quadrature error is reduced and the phase alignment accuracy is raised.

As shown in Figure 2b, the readout circuit is composed of a closed-loop drive circuit and an open-loop detection circuit. The drive circuit employs a PLL to achieve continuous frequency tracking and low frequency variation [33]. The PLL output passes through a resistance divider and then excites the device. Since the amplitude of the PLL output is constant, the output of the divider is invariant. If the temperature effect is not considered, the drive displacement is constant. This scheme eliminates the need for the traditional amplitude control circuit. As for the interface, the capacitance change of the gyro device is read by a trans-impedance amplifier (TIA) instead of a switched-capacitor amplifier or a charge amplifier due to its merits of low power, low noise, low capacitive load and low Q load. The analog output is quantized by a 16-bit Analog-to-Digital Converter, wherein the high resolution helps to reduce the quantization noise.

## 4. Drive Mode Circuit

**Figure 3:**
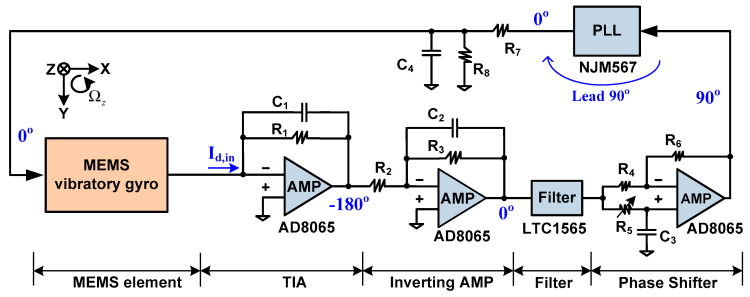
Closed-loop drive circuit based on PLL.

As shown in Figure 3, the drive circuit consists of a TIA, inverting amplifier, low-pass filter, phase shifter, PLL, resistance divider and passive filter. The inverting amp follows the TIA due to the anti-phase relationship between the actuation voltage and the TIA output. Considering the mechanical noise of the gyro, the filter LTC1565 [34] is connected behind the inverting amp. The PLL chip NJM567 [35] exhibits good frequency stability and excellent frequency tracking, whose center frequency is configured by an off-chip tunable resistor. Since the PLL output leads its input by 90° when the PLL locks, a phase shifter is inserted in front of the PLL to compensate the 90° phase shift. In addition, the phase shifter also compensates the slight phase shift introduced by the TIA, the inverting amp and the filter.

In an ideal condition, the relative phase of each node in the drive loop is shown in Figure 3. Considering the gyro’s nonlinearity and the feed-through of the actuation voltage, the PLL output is divided to a suitable value. Moreover, the actuation voltage is smoothed by a passive filter to reduce its harmonic components. As depicted in Figure 3, the positive terminal of the TIA is grounded, which leads to a virtual ground of the corresponding terminal in the gyro. Hence, a parasitic capacitor in the gyro is bypassed to ground, which further reduces the feed-through of the excitation voltage. In order to maintain stability, capacitors C_1_ and C_2_ are laid across the feedback branch of the TIA and the inverting amp, respectively.

### 4.1. Low Noise TIA

**Figure 4:**
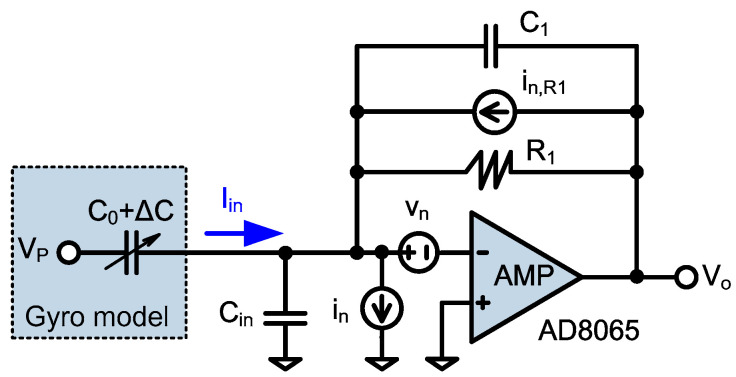
Low noise trans-impedance amplifier (TIA) with noise model.

Regarding the ultra-high Q value of the drive mode, the mechanical noise of the gyro can be negligible relative to the electrical noise. Meanwhile, the TIA, as the first stage in the drive circuit, dominates the electrical noise contribution and needs a low noise design. The TIA with noise model is shown in Figure 4. The noise characteristics of the amp are characterized by the input equivalent noise current i_n_ and the input equivalent noise voltage v_n_. The capacitance change ΔC represents the vibration displacement of the gyro while the output current I_in_ stands for the vibration velocity. The current to voltage conversion is realized by the feedback resistor R_1_, whose noise is modeled by i_n,R1_. The feedback capacitor C_1_ includes the stray capacitors, and its impedance value is much larger than R_1_. The input capacitor C_in_ contains the static capacitance between the mass and electrode, the gyro’s pad capacitance and the PCB trace parasitic capacitance.

When the impedance value of C_in_ is much larger than the equivalent input impedance of the TIA, the input current I_in_ mostly flows into the input terminal of the TIA. Hence, the transfer function is expressed by:

(1) $$\frac{V_{o}\left(s\right)}{I_{\mathrm{in}}\left(s\right)}\;=\;-R_{1}\frac{1}{{1+\mathrm{sC}}_{1}R_{1}}$$

Taking C_1_ = 0.5 pF and R_1_ = 1 MΩ, the 3 dB cut-off frequency of the TIA is about 0.3 MHz, which is much larger than the signal bandwidth and will not affect the signal processing. As for the low-noise amp AD8065 [36], i_n_ is 0.6 fA/√Hz, and v_n_ is 7 nV/√Hz. The total input equivalent noise is [37]:

(2) $$i_{\mathrm{in},\mathrm{eq},\mathrm{noise}}\;=\;\sqrt{{i_{n}}^{2}\;+\;\frac{4\mathrm{kT}}{R_{1}}\;+\;{\left(\frac{v_{n}}{R_{1}}\right)}^{2}\;+\;\frac{{\left(v_{n}{2\pi f}_{d}C_{\mathrm{in}}\right)}^{2}}{3}}$$

Herein, f_d_ is the signal frequency. Thanks to the ultra-low noise characteristic of the AD8065 and ultra-high resistance of the feedback resistor R_1_, the value of the first three terms of Equation (2) is small, and the fourth term dominates. Hence, reducing the input capacitance C_in_ is crucial. The following measures can be taken: (1) selecting the small package chip of the AD8065; (2) placing the AD8065 next to the gyro as close as possible to reduce the trace line in the PCB; (3) digging out the grounded copper near the trace, which connects the gyro and the inverting terminal of the AD8065.

### 4.2. Phase Shifter

**Figure 5:**
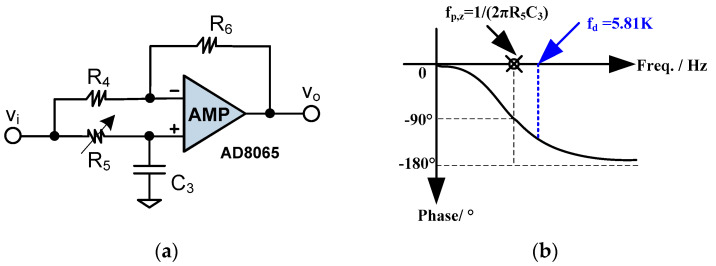
(**a**) Phase shift circuit with tunable resistor; (**b**) the phase frequency response.

In order to compensate for the leading 90° phase caused by the PLL, the phase shifter needs to provide a lagging 90° phase. Besides, the phase shifter must make up the slight phase shift introduced by the TIA, the inverting amp and the filter. The phase shifter is based on the amplifier of AD8065, as illustrated in Figure 5a. The resistors R_4_ and R_6_ form the negative feedback, while R_5_ and C_3_ constitute the tunable phase shift mechanism. 

Assuming the amplifier is ideal, using the “virtual short” and “virtual open” characteristics, we get:

(3) $$\frac{\frac{1}{{\mathrm{sC}}_{3}}v_{i}\left(s\right)}{\frac{1}{{\mathrm{sC}}_{3}}\;+\;R_{5}}-\frac{v_{i}\left(s\right)-\frac{\frac{1}{{\mathrm{sC}}_{3}}v_{i}\left(s\right)}{\frac{1}{{\mathrm{sC}}_{3}}\;+\;R_{5}}}{R_{4}}R_{6}\;=\;v_{o}(s)$$

If R_4_ = R_6_, then:

(4) $$\frac{v_{o}\left(s\right)}{v_{i}\left(s\right)}\;=\;\frac{1-{\mathrm{sR}}_{5}C_{3}}{1\;+\;{\mathrm{sR}}_{5}C_{3}}$$

Thus, the pole and zero coincide. The amplitude frequency response is a constant 0 dB, while the phase frequency response is:

(5) ∠vo(f)vi(f) = −2arctan(f12πR5C3)

The phase frequency response curve is shown in Figure 5b. Since the phase shifter needs to provide a phase shift slightly greater than 90°, the pole/zero should be a little lower than the resonant frequency of 5.81 KHz. If the pole/zero are set to 4.8 KHz and the R_5_ is 10 KΩ (for a 20 KΩ tunable resistor), then the C_3_ is calculated as 3.3 nF.

### 4.3. PLL

**Figure 6:**
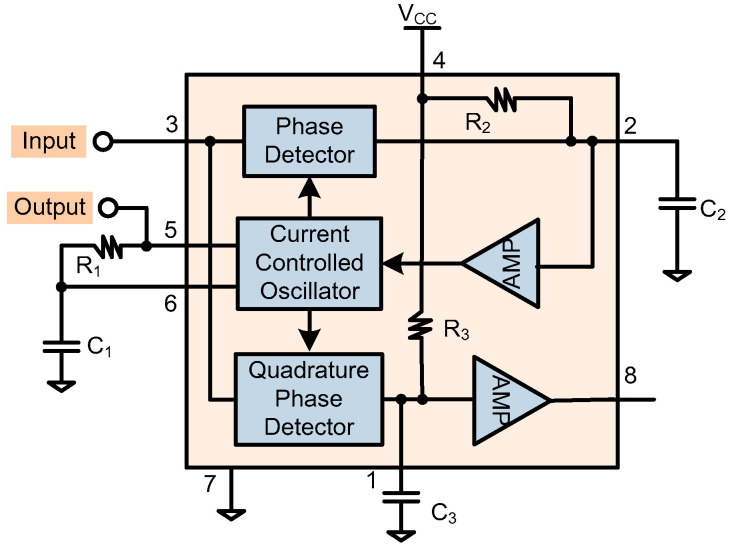
The PLL circuit based on NJM567, with pin 3 as input and pin 5 as output [35].

The PLL is based on the chip of NJM567. The circuit is shown in Figure 6 [35]. Pin 3 is used as the input, and pin 5, as the output, which is a rectangular wave and exhibits a leading 90° phase compared with pin 3 when PLL locked. Note that the output pin 8 does not function well. The PLL center frequency is set by R_1_ and C_1_, and the frequency range is 0.01 Hz–500 KHz. The frequency is set by the equation f_0_ = 1/(1.07 × R_1_ × C_1_). In light of the 5.81 KHz resonant frequency, if the C_1_ is set to 15 nF, then the R_1_ is derived as 10.6 KΩ (for a 20 KΩtunable resistor).

The maximum capture bandwidth is 14% × f_0_, which is set by the capacitor C_2_ and the PLL input amplitude [35]. If the curve of f_0_ × C_2_ = 1.3 × 10^3^ (Hz-μF) is selected and then C_2_ is set to 0.22 μF, a minimum input amplitude of 200 mV_rms_ is required. In this situation, a capture bandwidth of 5.4–6.2 KHz is calculated and it is large enough to resist the resonant frequency variation induced by the environmental disturbance.

## 5. Sense Mode Circuit

**Figure 7:**
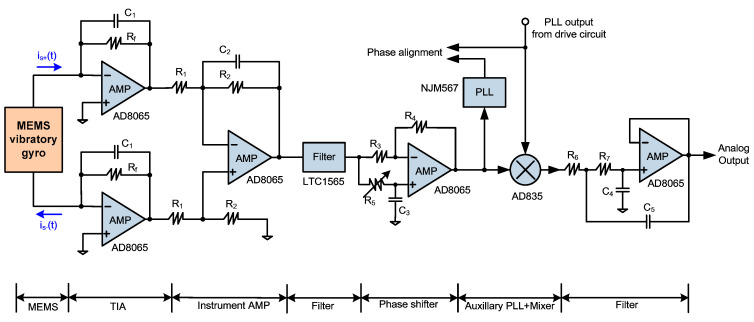
Open-loop detection circuit based on quadrature error and auxiliary PLL.

The overall detection circuit is shown in Figure 7. It consists of a differential TIA, instrumentation amplifier, low-pass filter, phase shifter, auxiliary PLL, mixer and active low-pass filter. Due to the large mechanical noise, the LTC1565 filter is inserted in the detection path to improve the signal-to-noise ratio. In order to achieve a 90° phase alignment between the quadrature error and the carrier, the phase shifter is inserted in front of the mixer to achieve a lagging phase shift of 0° to −90°. The auxiliary PLL is realized by NJM567. When the auxiliary PLL output and the carrier are 180° phase aligned, the quadrature error is simultaneously aligned with the carrier by 90°, and the Coriolis is concurrently in synchronization with the carrier, which leads to a correct coherent demodulation! The demodulator is based on AD835 [38], and the low-pass filter is based on the second-order Butterworth Sallen–key type filter [39].

### 5.1. C/V Conversion Circuit

**Figure 8:**
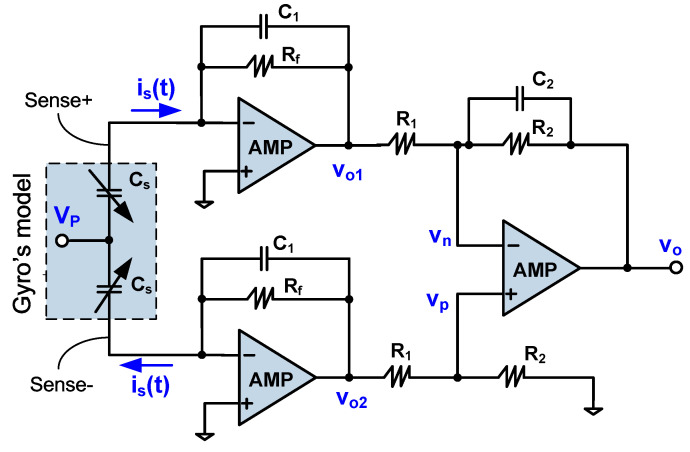
Overall C/V conversion circuit based on low-noise TIA.

The gyro in the sense mode can be equivalent to a pair of differential capacitors C_s_, as shown in Figure 8. The capacitance change ΔC_s_ corresponds to the vibration displacement of the mass and generates the current i_s_(t) when the polarization voltage V_P_ is applied to the common terminal of the differential capacitors. The current is V_P_ × d(ΔC_s_)/dt. In addition, “sense+” and “sense−“ electrodes need to be grounded to minimize the parasitic capacitance, so each electrode is followed by a TIA whose non-inverting terminal is grounded. In order to suppress high frequency noise and prevent oscillation, the small capacitors C_1_ and C_2_ are added in the feedback branches, respectively.

Considering the drive-mode resonant frequency is 5.81 KHz, only the low frequency response of the C/V conversion circuit needs to be paid attention to. As shown in Figure 8, the upper TIA output is $v_{o1}\left(t\right)\;=\;-i_{s}\left(t\right)\times R_{f}$, and the lower is $v_{o2}\left(t\right)=\;i_{s}\left(t\right)\times R_{f}$. Assuming the second stage amp is ideal, using the virtual break characteristic of the amplifier, we have $v_{p}\left(t\right)=v_{o2}\left(t\right)\times R_{2}/\left(R_{1}+R_{2}\right)$, and $v_{n}\left(t\right)=\left(v_{o1}\left(t\right)-v_{o}\left(t\right)\right)\times R_{2}/\left(R_{1}+R_{2}\right)+v_{o}\left(t\right)$. Then, by applying the virtual short feature and letting $v_{n}\left(t\right)\approx v_{p}\left(t\right)$, we get the output:

(6) $$v_{o}(t)\;=\;\frac{R_{2}}{R_{1}}\left(v_{o2}\left(t\right)-v_{o1}\left(t\right)\right)\;=\;2i_{s}{(t)R}_{f}\frac{R_{2}}{R_{1}}$$

In the actual design process, the following values were taken: R_f_ = 1 MΩ, C_1_ = 0.5 pF, R_1_ = 100 Ω, R_2_ = 10 KΩ and C_2_ = 1 pF.

### 5.2. Coherent Demodulator

**Figure 9:**
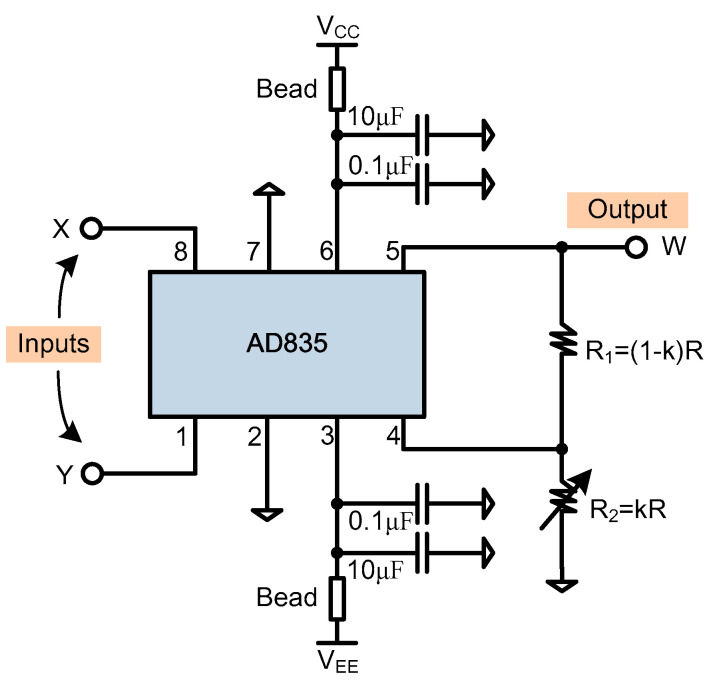
The coherent demodulator (Mixer) based on AD835, where X and Y are the input and W is the output [38].

The demodulator is shown in Figure 9, which is based on the AD835 chip [38]. The two inputs are connected to pin 8 and pin 1, and the output, to pin 5. The chip adopts a dual power supply. In order to make the output have a good reference, the lower end of the resistor R_2_ needs to be grounded, as shown in the Figure 9. If the total resistance mounted on the output is R and the small resistor R_2_ is k × R, then the large resistor R_1_ is derived as (1−k) × R. The function from the inputs to the output is [38]:

(7) $$W\;=\;\frac{\mathrm{XY}}{\left(1-k\right)U}$$

Herein, U is a trimming voltage, and the typical value is 1.05 V. In order to realize the simplified expression of W = XY, let (1 − k) × U = 1, then k = 0.0476. That is, R_1_ = 20 × R_2_. Let R_2_ = 200 Ω; then, R_1_ = 2 KΩ.

### 5.3. Low Pass Filter

**Figure 10:**
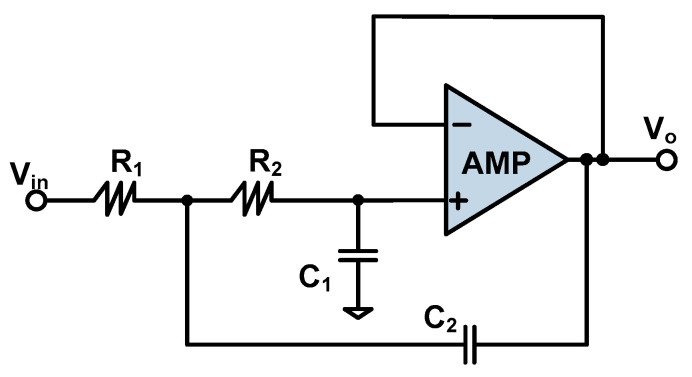
Second-order Butterworth active low-pass filter (Sallen–Key type) [40].

The filter uses a Butterworth type filter because it has good low-pass characteristics and has the maximum flatness in the passband [39]. The filter is of the second-order form and the Sallen–Key scheme, as shown in Figure 10.

The filter gain is set to 1. According to KCL (Kirchhoff’s current law) and KVL (Kirchhoff’s voltage law), the transfer function of the filter is

(8) $$\frac{V_{o}\left(s\right)}{V_{\mathrm{in}}\left(s\right)}\;=\;\frac{1}{{1\;+\;C}_{1}\left(R_{1}{\;+\;R}_{2}\right){s\;+\;R}_{1}R_{2}C_{1}C_{2}s^{2}}$$

Let the normalization coefficients be:

(9) $$a_{1}\;=\;\omega_{C}C_{1}{(R}_{1}{\;+\;R}_{2})$$

(10) $$b_{1}\;=\;\omega_{C}{}^{2}R_{1}R_{2}C_{1}C_{2}$$

Herein, $\omega_{C}$ is the corner frequency of the Butterworth filter. If the values of C_1_ and C_2_ are given, the values of R_1_ and R_2_ can be calculated:

(11) $$R_{1,2}\;=\;\frac{a_{1}C_{2}\pm \sqrt{a_{1}{}^{2}C_{2}{}^{2}-{4b}_{1}C_{1}C_{2}}}{{4\pi f}_{C}C_{1}C_{2}}$$

Since the resistance value is a real number, the root number must be greater than 0, so

(12) $$C_{2}\geq C_{1}\frac{{4b}_{1}}{a_{1}{}^{2}}$$

Since the maximum bandwidth of the input angular velocity is 80 Hz, the corner frequency of the Butterworth filter needs to be greater than 800 Hz. If the corner frequency is set to 1 KHz and use is made of the Butterworth table [40], the optimal values of the normalized coefficients a_1_ and b_1_ can be obtained: a_1_ = 1.3617, b_1_ = 0.618. If the value of C_1_ is set to 10 nF, then C_2_ must be larger than 13.33 nF. By setting the value of C_2_ to 15 nF and substituting these parameter values into Equation (11), we get R_1_ = 7.22 KΩ and R_2_ = 14.45 KΩ.

## 6. Experimental Results

**Figure 11:**
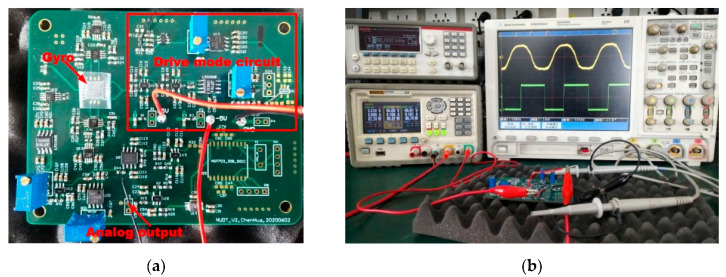

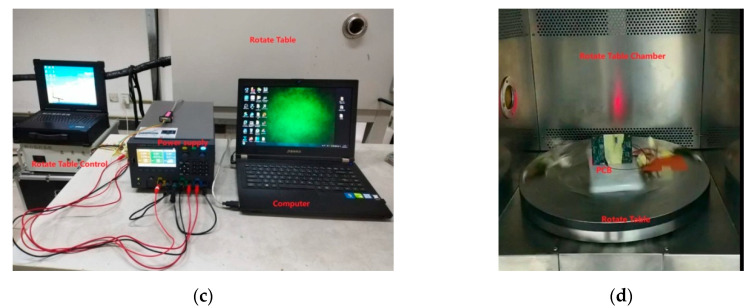
Functional and performance measurement. (**a**) PCB board; (**b**)functional test; (**c**) rotating table test; (**d**) rotating table chamber and setup of the PCB.

The readout circuit of the MEMS vibratory gyro was implemented on the PCB, as shown in Figure 11a. The upper half is the drive mode circuit, the lower half is the sense mode, and the middle is the positive supply, negative supply and ground. The gyro device is under the plastic cover. The TIAs of the drive and sense mode are closely next to the gyro to reduce the trace parasitic capacitance [36,37]. Additionally, the TIAs in the sense mode are laid symmetrically to achieve high performance. Since the traces connecting the gyro and the TIA are sensitive, the grounded coppers are dug out to reduce the feed-through. The PLL and Analog-to-Digital Converter are located at the corner of the PCB to prevent them from contaminating the rest blocks, especially the TIAs.

The function and phase alignment test is shown in Figure 11b. The measurement equipment consists of a power supply, RIGOLDP1308A; a function generator, KEITHLEY3390; and an oscilloscope, AgilentDSO7034B. The rotating table measurement is illustrated in Figure 11c. The test equipment includes a low-noise power supply, KEYSIGHTE36313A; a single-axis angular rate temperature-controlled rotating table, Hangzhou AOBOSLT-01V1-100C; and its control system. Since the gyro is a Y-axis angular rate gyro, the PCB was erected and fixed on the rotating table as depicted in Figure 11d. The power and signal lines on the PCB were connected to the outside by the slip ring of the rotating table.

### 6.1. Function and Phase Alignment Measurement

**Figure 12:**
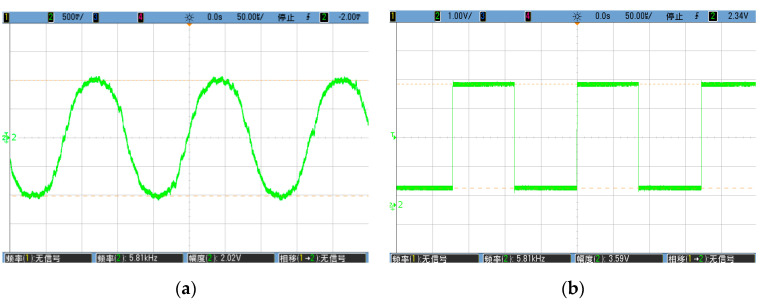

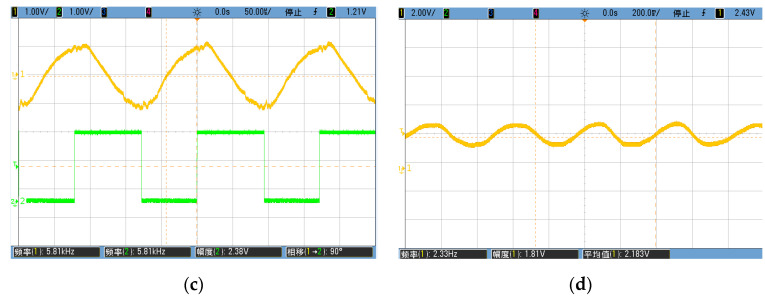
(**a**) Quadrature error of the detection path; (**b**) Waveform of the quadrature error after passing through the auxiliary PLL; (**c**)Waveform of the quadrature error and the demodulated carrier, phase aligned at 90°; (**d**) Analog output when the PCB was rotated by hand.

Firstly, the gyro was allowed to remain static and the drive mode circuit was opened. The function generator was used to excite the gyro, and the output of the inverting amp was carefully measured to find the exact resonant frequency of the drive mode. Secondly, the tunable resistor of the phase shifter was adjusted, letting its output phase lag the excitation voltage by 90°. The PLL free-running frequency was set near to the drive-mode resonant frequency. Thirdly, the drive mode circuit was closed and it was ensured that the stable oscillation was built up. The output of the filter was measured in the sense mode to identify the quadrature error, as shown in Figure 12a. Its frequency was 5.81 KHz, and its amplitude was 1.01 V. It passed through the phase shifter and the auxiliary PLL to become a low jitter signal as illustrated in Figure 12b. Fourthly, the tunable resistor of the sense-mode phase shifter was adjusted and the auxiliary PLL output and the carrier were set in 180° alignment. Simultaneously, the two inputs of the mixer were in 90° alignment, as shown in Figure 12c, wherein the yellow is the phase-shifted quadrature error and the green is the carrier. Finally, the PCB was rotated along the Y-axis by hand to simulate an angular rate input, and the demodulated result is depicted in Figure 12d. The peak-to-peak swing was about 2 V, which shows that the gyro and its readout worked properly and were very sensitive.

### 6.2. Scale Factor Measurement

**Figure 13:**
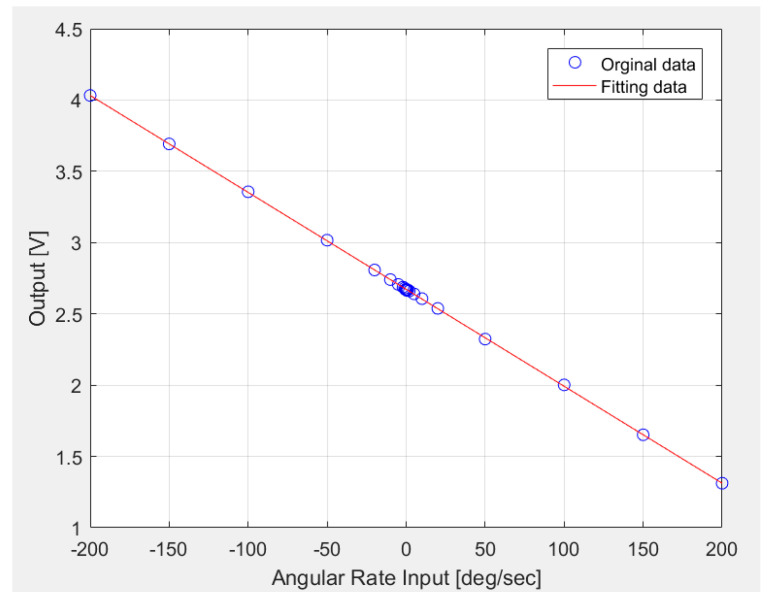
Scale factor of the MEMS gyroscope.

According to the IEEE gyro test standard, the gyro was set, the power was turned on, half an hour was allowed to elapse, and the angular rate was controlled at 0°/s, ± 0.1°/s, ± 0.2°/s, ± 0.5°/s, ± 1°/s, ± 2°/s, ± 5°/s, ± 10°/s, ± 20°/s, ± 50°/s, ± 100°/s, ± 150°/s and ± 200°/s. The digital outputs were collected by a data acquisition system, and its sample rate was 65 Hz. At each angular rate point, the output was acquired for 10 s and then an average function was used to obtain the mean value. The least squares method was used to fit the original data. By using the MATLAB function of polyfit (x, y, 1), the scale factor was plotted as shown in Figure 13. The fitted result is Y = −0.0068 × X + 2.6721 [V], so the scale factor is 6.8 mV/°/s and the zero offset is 2.6721 V.

### 6.3. Bias Instability Measurement

**Figure 14:**
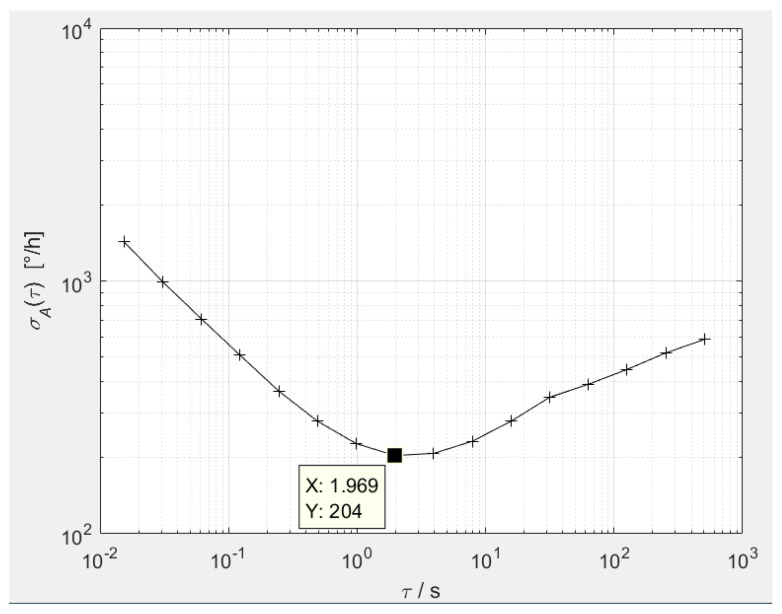
Bias instability of the MEMS gyroscope.

The input angular rate was set to 0°/s, and the digital output was recorded for two hours. Based on the Allan variance equation [41], τ was set at 1/65, 2/65, 4/65, 8/65, 16/65, 32/65, 64/65, 128/65, 256/65, 512/65, 1024/65, 2048/65, 4096/65, 8192/65, 16384/65 and 32768/65 s, respectively, and the Allan variance calculation was performed. Finally, the Allan variance curve was plotted as shown in Figure 14. From 0.01 to 1 s, there is an angular random walk with a slope of −1/2, which is determined by the white noise of the angular rate, that is, the Brown noise of the gyro device. From 2 to 4 s, there is the bias instability, which is determined by the 1/f (flicker) noise of the angular rate. At 5 s and above, a rate random walk with a slope of +1/2 appears, which is determined by the white noise of the angular acceleration. On the whole, the curve presents a flat bottom, but the interval is narrow, which is caused by the excessively large Brown noise of the gyro and the white noise of the angular acceleration.

To explain the Allan curve and guide the next optimization, it is necessary to analyze the limiting factors of the bias instability. In a sense, the Leeson formula [42] describing the phase noise of the oscillator can guide the zero-bias optimization of the gyroscope. To reduce the bias instability, it is necessary to increase the gyro’s Q value, lower the resonant frequency, and reduce the electrical thermal and 1/f noise. As shown in Figure 14, the bias instability is 204°/h, which is two orders of magnitude higher than the latest research level [6,11,18]. The bias instability performance is not good. The limiting factors may be (1) the Q value of the sense mode being too low, (2) the 1/f noise of the readout circuit being high, or (3) the mechanical noise of the gyro device being large. Other minor factors may be (1) the excitation voltage in the drive mode being quasi-square and the C/V conversion circuit being single-ended, exhibiting more feedthrough; (2) the amplitude stability of the drive displacement in the drive mode not being high enough; or (3) the amplitude of the quadrature error in the sense mode being too large, leading to the detection link gain being too small and the electrical signal-to-noise ratio being too low.

The next optimization measures are (1) selecting a high Q and low-noise gyro device; (2) using techniques such as chopping and correlated double sampling to reduce the electrical 1/f noise; (3) adding an amplitude-level-control circuit, adopting the sine-wave actuation and employing a differential C/V circuit; and (4) performing a partial quadrature error cancellation in the sense mode to relieve the low gain pressure.

## 7. Conclusions

This paper presents a novel readout technique based on aligning the quadrature error and demodulation carrier at 90° by using a tunable phase shifter on the detection path. The method can demodulate angular velocity, regardless of the relationship between the drive position and the Coriolis displacement. Moreover, by using an auxiliary PLL, the phase jitter of quadrature error is reduced, and the accuracy of the phase alignment is improved. Through detailed analysis and careful design, a complete drive circuit and detection circuit were finally realized. The experimental results showed that the readout circuit functioned well. The scale factor of the MEMS gyro was 6.8 mV/°/s, and the bias instability was 204°/h. The new coherent demodulation method can also be applied to other vibratory MEMS gyroscopes.

## 8. Patents

Hua Chen, Yanqing Zhong andYubao Fan—“Adaptive phase alignment module and method, and measurement and control circuit of vibrating gyroscope”, Chinese patent, application no.: 201910034788.0, application date: 2019.01.14, status: disclosure.

Hua Chen, Qiangtao Lai, Guiliang Guo, etc.—“A Closed-loop PLL-based Driving Circuit for MEMS gyroscope”, Chinese patent, application no.: 201610357769.8, application date: 2016.05.26, status: disclosure, reexamination.

## Figures and Tables

**Table 1:** MEMS gyro’s parameters.

Parameters	Values
Drive-mode resonant frequency	5.817 KHz
Drive-mode Q value	35,000
Sense-mode resonant frequency	6.05 KHz
Sense-mode Q value	500
Vacuum level	100 mTorr
Work mode	Mode splits
DC polarization voltage	5–10 V
Quadrature error ratio	0.22
